# Patient navigation services for cancer care in low-and middle-income countries: A scoping review

**DOI:** 10.1371/journal.pone.0223537

**Published:** 2019-10-17

**Authors:** Milena Dalton, Emily Holzman, Erica Erwin, Sophia Michelen, Anne F. Rositch, Somesh Kumar, Verna Vanderpuye, Karen Yeates, Erica J. Liebermann, Ophira Ginsburg

**Affiliations:** 1 Department of Population Health, NYU School of Medicine, New York, New York, United States of America; 2 New York University College of Global Public Health, New York, New York, United States of America; 3 Better Outcomes Registry & Network (BORN) Ontario, Ottawa, CA; 4 Independent Researcher, New York, New York, United States of America; 5 Johns Hopkins Bloomberg School of Public Health, Baltimore, Maryland, United States of America; 6 Jhpiego, Baltimore, Maryland, United States of America; 7 Korle Bu Teaching Hospital, Accra, Ghana; 8 New York University Rory Meyers College of Nursing, New York, New York, United States of America; 9 Perlmutter Cancer Center, NYU Langone Health, New York, New York, United States of America; University Lyon 1 Faculty of Dental Medicine, FRANCE

## Abstract

**Background:**

Nearly 70% of all cancer deaths occur in low- and middle-income countries (LMICs) and many of these cancer deaths are preventable. In high-income countries (HICs), patient navigation strategies have been successfully implemented to facilitate the patient’s journey at multiple points along the cancer care continuum. The purpose of this scoping review is to understand and describe the scope of patient navigation interventions and services employed in LMICs.

**Methods:**

A systematic search of published articles was conducted including Medline, Biosis, Embase, Global Health, and Web of Science. Articles were examined for evidence of patient navigation interventions used in cancer care in LMICs. Evidence was synthesized by navigation service provided and by type of outcome.

**Results:**

Fourteen studies reported on patient navigation interventions in cancer care in low-income and middle-income countries in Asia, South America, and Africa. Most studies reported on women’s cancers and included navigation interventions at most points along the cancer care continuum i.e. awareness, education, screening participation, adherence to treatment and surveillance protocols.

**Conclusion:**

Few studies report on cancer patient navigation in LMICs. With the use of an implementation science framework, patient navigation research can explore a broader range of outcomes to better evaluate its potential role in improving cancer control in LMICs.

## Introduction

Nearly 70% of all cancer deaths occur in low- and middle-income countries (LMICs) (1). The number of new cases and subsequent cancer deaths in LMICs is expected to grow substantially in the coming decades, due in part to population growth, shifts in demographics and exposures to known risk factors, in keeping with the rise of other non-communicable diseases (NCDs) [1, 2]. However, many such cancer deaths are premature and preventable [1]. Survival from cancer is strongly associated with geography, with patients in LMICs faring worse than those living in upper middle-income and high-income countries (HICs) [3, 4]. Key factors contributing to global disparities in cancer mortality and survival include differences in the proportion of patients diagnosed with later-stage disease, poor access to high quality, affordable treatment, fragile or fragmented health systems, as well as sociocultural, geographic, and financial barriers [1, 5, 6]. To address some of these challenges in HICs, patient navigation services should be included as best practices in comprehensive cancer care [7].

The inclusion of patient navigation services in HICs is associated with improvements in access to timely diagnosis, treatment, and follow-up [8], increases in screening participation [9], and treatment adherence [10], especially for vulnerable and marginalized populations [11]. The main purpose of incorporating patient navigation services into cancer care is defined by Wells et al., as reducing “delays in accessing continuum of care services, with an emphasis on the timeliness of diagnosis and treatment and a reduction in number of patients lost to follow up” [12]. This definition of navigation comprises a number of activities including coordinating care, facilitating linkages to follow-up services, and reducing or eliminating barriers to cancer care (Table 1).

**Table 1:** Patient navigation services in high-income countries.

• Coordinating provider appointments to ensure timely delivery of diagnostic and treatment services [11–15].
• Maintaining communication with patients, families and the health care providers to monitor patient satisfaction with the cancer care experience [11, 12, 16].
• Ensuring the availability of appropriate medical records at scheduled appointments [11, 13].
• Arranging language translation or interpretation services [11, 12].
• Facilitating financial support and helping to complete paperwork [11, 12, 17].
• Arranging transportation and/or child/elder care [11, 13, 17].
• Attending appointments with patients [12].
• Facilitating linkages to follow-up service [11, 14, 18, 19].
• Providing psychosocial support [12].

However, despite progress in the incorporation and value of patient navigation services in HICs, particularly among underserved populations, few cancer care facilities offer patient navigation services in LMICs. Cancer patients in LMICs often forego or discontinue treatment, leading to disproportionately lower survival rates and quality of life when compared to countries’ higher income counterparts [20]. Sociocultural, financial, health system and knowledge barriers keep many in LMICs from accessing appropriate cancer care services.

To contextualize patient navigation as a growing service for cancer care in LMICs, it is important to understand the particular access challenges that patient navigation in cancer care seeks to assist patients in overcoming. Patients face health system barriers as well as sociocultural barriers. One of the greatest barriers to accessing cancer care in LMICs is lack of geographic access to health facilities and lack of well-trained care providers with resulting poor distribution of services across a region or country [5]. This is particularly the case for specialized and multimodality care, which is required for most patients with cancer [21]. Even where specialized services are available, they are often dependent on government funding, not consistently available or affordable, and require complex coordination of care across a fragmented health system [20, 22, 23].

In addition to a number of health systems barriers, there are sociocultural factors that can affect patients’ decisions or ability to seek cancer care [24]. Cancer-related fears, such as prevailing ideas that cancer could be contagious, a punishment or type of “divine retribution,” or inevitably lead to death are potential barriers to seeking cancer care [25]. Likewise, the concern that cancer surgery might result in deformity, (such as that associated with a mastectomy) and that this in turn will lead to divorce and/or family abandonment is pervasive in some communities. As a result, women in particular might delay seeking care in the allopathic health system in favor of traditional healers [26]. To address these challenges, some patient navigation initiatives for cancer care have been proposed and implemented in LMICs to remove these barriers for those seeking care across multiple health care platforms (primary, secondary, tertiary), through various types of health care workers and communication channels, and in many different contexts.

The purpose of this scoping review is to understand the scope of patient navigation interventions and services employed in LMICs to assist patients in overcoming social, cultural, and structural barriers, to navigate care pathways, and to receive timely and appropriate care. To the best of our knowledge, this is the first such review.

## Methods

### Search strategy

For this review, the research team designed a comprehensive search strategy in consultation with a medical education librarian. As the aim for this review was to review the literature on patient navigation interventions for cancer care in low-and middle-income countries, identify research gaps and summarize research findings, the team conducted a scoping review. [27] A systematic search of Medline, Biosis, Biological abstracts, Embase, Global Health, Web of Science, Scielo, and Scopus databases was conducted, followed by a comprehensive search of the grey literature, which included UN agency websites and Google Scholar. The team followed PRISMA guidelines to conduct this scoping review, and keywords were developed from a number of thesauri including MeSH databases and EMTREE for appropriate headings and text words [28]. Due to the various types of health workers conducting patient navigation services in LMICs, search terms for health workers were identified through WHO’s health worker classification [29]. Key search terms included “patient navigation” and “cancer” and “developing countries” or “low- and middle-income countries”. A number of restrictions were placed on the search criteria to include only English language articles and articles published from 2003 to the present. Additional articles were extracted from the reference lists of selected systematic reviews. Finally, an e-hand search of the most-identified journals was conducted to ensure that all relevant articles were captured in the search. After this initial search, two more articles were identified in the review process and added to the final number of articles included. The search concluded on December 6, 2018, and the complete strategy is described in full in the S1 Table.

### Inclusion and exclusion criteria

**Fig 1:**
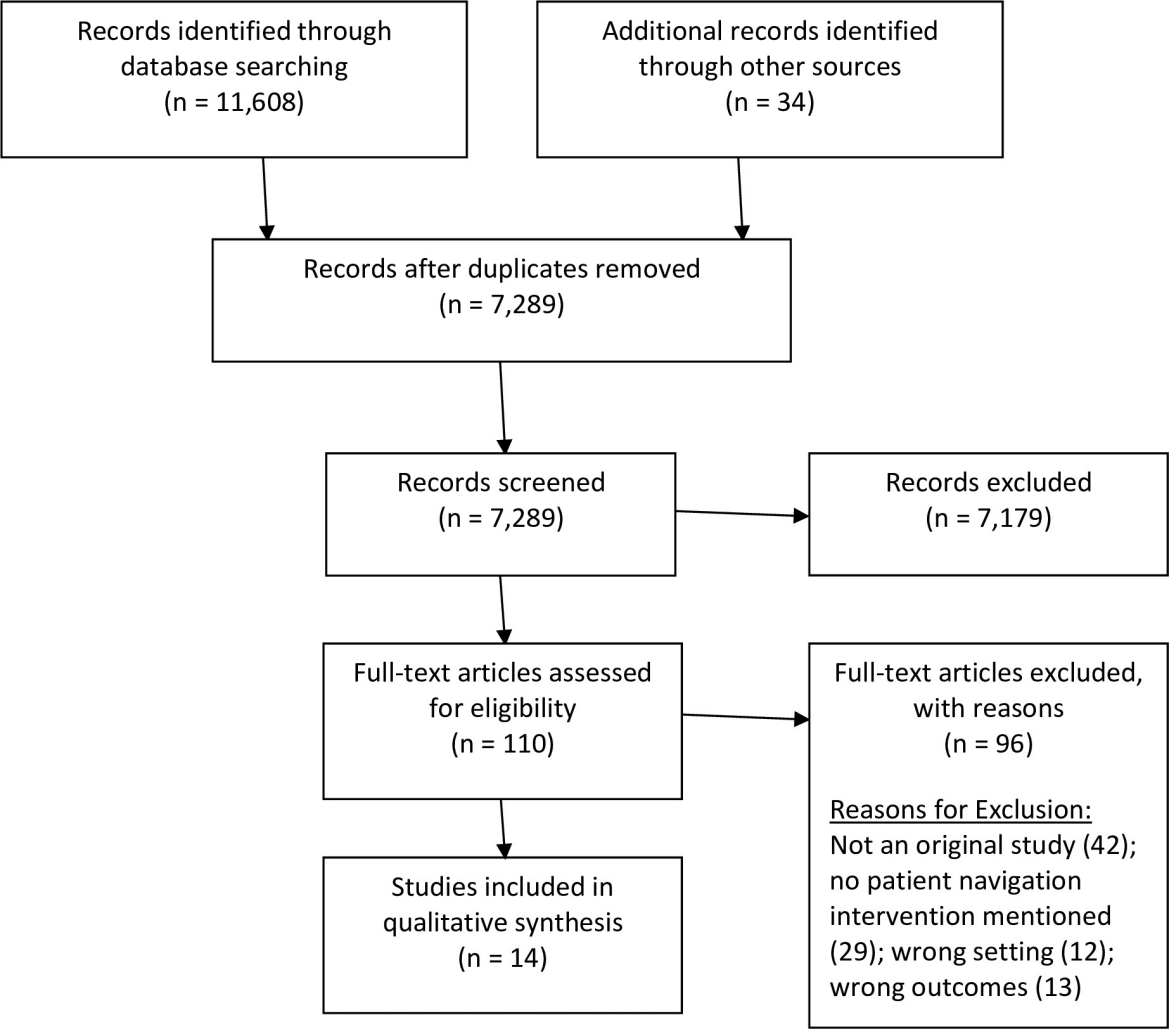
PRISMA flow diagram.

This review includes studies that contained information about cancer care and a patient navigation intervention in an LMIC. The research team used the World Bank’s criteria to determine the income status of the countries identified in the search corresponding to the year of data collection. Results from the search were refined to include only original research studies, studies that focused on LMICs, and studies that mentioned patient navigation or patient navigation services. The definition of patient navigation services is from the current literature on patient navigation services offered in HICs (Table 1) as well as the patient navigation services defined in Wells et al.[12] and Ginsburg et al. [6]. Fig 1 displays the PRISMA flow diagram, which demonstrates the review of 110 texts, resulting in 14 studies included in the qualitative synthesis.

### Data processing

Two teams of two authors reviewed all study abstracts and titles captured from the database search. Each team reviewed the full text for 55 identified articles, totaling 110 articles. The teams compared results within each team and resolved discrepancies by discussion or with the involvement of a third reviewer. For the full-text review, the research team maintained a comprehensive log for excluded studies and noted the reasons for exclusion (Fig 1).

### Data extraction and assessment of quality

The research team designed a comprehensive data extraction form and tested the form with five articles. The data extraction form included the various types of patient navigation services defined from Ginsburg et al. [6] and considered the various types of clinical, process, and implementation outcomes [30] from each study. Two teams of two authors performed the data extraction and each team reviewed their work internally. Recorded study features included authors, year of publication, country of study, and the data extraction form and template collected specific data points relevant to cancer care and patient navigation services in LMICs. The study team assessed each article included in the review for rigor, sample size, and study design. No articles were excluded from the review based on these characteristics.

## Results

### Study characteristics and populations

Fourteen studies met the criteria for extraction. All 14 studies were quantitative studies. The region (as defined by WHO) with the highest number of studies was the Region of the Americas (five studies, 35.7%), followed by the Southeast Asia Region (four studies, 28.6%). During the time of data collection, the countries included were primarily upper-middle income countries (eight studies, 57.1%), as defined by the World Bank. Five studies (35.7%) included lower-middle income countries and one study (7.1%) included a low-income country. All included publications that were published in 2012 or later, and the study setting was mainly urban (eight studies, 66.7%) (Table 2).

**Table 2:** Study characteristics.

		n = 14(%)
Year of publication		
	2012–2018	14 (100%)
WHO Region		
	African	1 (7.1%)
	Eastern Mediterranean	2 (14.3%)
	Western Pacific	2 (14.3%)
	Region of the Americas	5 (35.7%)
	South-East Asian	4 (28.5%)
Continent		
	Africa	1 (7.1%)
	North America	2 (14.3%)
	South America	3 (21.4%)
	Asia	8 (57.1%)
Study Type		
	Before and after study	1 (7.1%)
	Population-based study	1 (7.1%)
	Retrospective cohort	1 (7.1%)
	Household survey	1 (7.1%)
	Not stated	1 (7.1%)
	Prospective cohort	2 (14.3%)
	Randomized control trial	3 (21.4%)
	Experimental design	4 (28.6%)
Study setting		
	Not stated	1 (7.1%)
	Peri-urban	2 (14.3%)
	Rural	3 (21.4%)
	Urban	8 (57.1%)
Patient navigation intervention duration		
	1–3 months	2 (14.3%)
	3–6 months	2 (14.3%)
	6–12 months	2 (14.3%)
	12 months+	4 (28.6%)
	Not stated	4 (28.6%)
Type of cancers		
	Bladder	1 (7.1%)
	Head and neck	1 (7.1%)
	Gastrointestinal, genitourinary, endocrine, hematologic, breast, other	1 (7.1%)
	Not stated	1 (7.1%)
	Cervical	2 (14.3%)
	Breast	8 (57.1%)

Studies primarily focused on breast cancer (eight studies, 57.1%), followed by cervical cancer (two studies, 14.3%) (Table 2). Among the 14 studies, the median sample size was 524 (range: 22–22,337). A majority of the studies assessed patients aged 18 and older (eight studies, 57.1%), while only two studies reported on those 18 and under.

### Types of patient navigation services

The patient navigation services outlined in the studies often included multiple interventions per study. The most common service offered was centered around counselling to assist patients in understanding their signs, symptoms or surgical procedures and education around primary and secondary prevention (nine studies, 64.3%) and facilitating linkages to follow-up services and support (nine studies, 64.3%). Eight studies included coordinating appointments (57.1%). Some of the other services in the studies included showing motivational and educational videos on mobile phones, providing tours of the clinics, spiritual support and more. The most common health workers to carry out patient navigation services were nurses (four studies, 28.6%). The most common way patient navigation services were offered were through individual and in-person sessions (ten studies, 71.4% and 9 studies, 64.3%, respectively) or through mHealth (mobile health) platforms or telephone calls (ten studies, 71.4%) (Table 3).

**Table 3:** Characteristics of patient navigation services.

		n = 14 (%)
Type of patient navigation services*		
	Ensuring availability of medical records	1 (7.1%)
	Strengthening family capacity to provide support	3 (21.4%)
	Maintaining communication with families, patients, and providers	3 (21.4%)
	Appointment reminders	4 (28.6%)
	Facilitating financial support and helping to complete paperwork	4 (28.6%)
	Arranging transportation and/or elder/child care	4 (28.6%)
	Other	5 (35.7%)
	Coordinating appointments	8 (57.1%)
	Counselling or education to ensure understanding of symptoms or signs	9 (64.3%)
	Facilitating linkages to follow-up services & support	9 (64.3%)
Type of health worker executing service**		1 (7.1%)
	Researcher	1 (7.1%)
	Psychosocial team	1 (7.1%)
	Patient navigator	1 (7.1%)
	Health professional	1 (7.1%)
	Doctor	1 (7.1%)
	Trained educators	3 (21.4%)
	Community health worker	4 (28.6%)
	Nurse	
Publicly- or privately-owned facilities		
	Not stated	3 (21.4%)
	Private	5 (35.7%)
	Public	6 (42.9%)
Level in the health system***		
	Primary	1 (7.1%)
	Secondary	1 (7.1%)
	Not applicable	2 (14.3%)
	Community	4 (28.6%)
	Tertiary	7 (50.0%)
Type of patient navigation session		
	Group	1 (7.1%)
	Not stated	3 (21.4%)
	Individual	10 (71.4%)
Communication channel****		
	Other	1 (7.1%)
	Print	5 (35.7%)
	In-person	9 (64.3%)
	mHealth & telephone calls	10 (71.4%)

*Multiple services are offered per study. Therefore, the total of n will not equal 14.

**One study used both a nurse and doctor to carry out PN services.

***One study assesses the navigation service at two levels of the health system.

****Multiple studies used multiple communication channels to deliver their intervention.

### Levels of health system and level of care

Patients accessed services mostly at the tertiary level (seven studies, 50.0%) of the health system in each country, followed by the community level (four studies, 28.6%) (Table 3). The type of health facility where the services were offered varied greatly and ranged from walk-in clinics to national and public hospitals. One study implemented a patient navigation intervention in the workplace. Many of the health facilities were publicly funded and government-owned (six studies, 42.9%). Most interventions occurred at the secondary prevention level (i.e. screening) and at the treatment and follow-up level (eight studies, 57.1%). Five studies (35.7%) occurred at the diagnosis level, 2 studies (14.2%) occurred at the primary prevention level and 2 studies (14.2%) at the supportive care level.

### Types of outcomes

Process, implementation and clinical outcomes were identified in the included articles. All studies showed some degree of positive effect of patient navigation interventions on the primary outcome measured. Twelve articles (85.7%) reported on process outcomes, which included coordination of appointments, follow-up and referrals, and the ability to overcome sociocultural and financial barriers. Twelve articles (85.7%) reported on implementation outcomes, which included knowledge and attitudes of patients and community members, acceptability of the intervention, uptake of the intervention by patients (reach), fidelity to the intervention, and more. Nine articles (64.3%) reported on clinical outcomes, which included screening rates, post-operative complications, patient retention (in treatments) and more. Interestingly, no articles reported that they were conducting implementation science, even though nearly all of them reported implementation outcomes. Table 4 describes the types of outcomes in each article, the primary outcome of the article, followed by the primary outcome results.

**Table 4:** Description of outcomes.

Author *Country*	Types of Outcomes	Primary Outcome	Primary Outcome Results
Ma GX (2012)	Clinical Process Implementation	The impact of a workplace intervention on increasing breast cancer screening rates	The workplace intervention increased screening rates via mammography from 10.3% to 72.6% in the intervention group. In the control group, screening rates decreased from 5.9% to 4.7% within the 6-month follow-up period.
*China*			
Ginsburg O (2014)	Process Implementation	Adherence (advice regarding a clinical appointment) for women with an abnormal CBE.	Adherence in arms A (smart phones without navigation) and B (smart phones plus patient navigation) versus the control arm was the same (53%). Women using smart phones plus patient navigation (Arm B) were significantly more likely to attend for proper care when compared to women who used smart phones without navigation (Arm A) (63% vs. 43%,) (p, .0001).
*Bangladesh*			
Chowdhury TI (2015)	Process Implementation	Feasibility of serious breast problem case-finding by community health workers using either paper or cellphone recording of basic individual patient data.	Of the women with breast abnormalities, four of these women were from the paper data collection group. One of these women followed up at the Amader Gram Breast Center. Six women who were in the motivational and navigational groups had breast abnormalities. Four of these women were seen at the breast center.
*Bangladesh*			
Nejad Z (2016)	Implementation	Determine and compare the caregiver strain index scores of breast cancer informal caregivers, before and after a patient-caregiver educational and telephone follow-up program.	Caregiver strain scores decreased for the intervention group, who received face-to-face education, telephone follow-up, and personal training from 8.3 ± 2 to 4.8 ± 2.3 post intervention.
*Iran*			
Li XQ (2016)	Clinical Process	Occurrence rate of postoperative complications (infection, haemorrhage, bedsore and malnutrition).	Rates of postoperative complications for the observation group, who received telephone follow-up, coordination with caretakers, assessment of living conditions and psychological comfort, were significantly lower than that of the control group. It was also found that length of hospital stay was also shorter, and patients were significantly more satisfied with the nursing service that they had received (P<0.05).
*China*			
Sajjad S (2016)	Process Implementation	Change in a Quality of life survey completed by patients (conducted at baseline [T1] and at sixth week of receiving chemotherapy [T2]).	Quality of Life (QoL) scores significantly increased in the intervention group who received patient education, face-to-face discussions with nurses, and telephone follow-up from nurses, when compared to the control group. These improvements were most significant at the T2 level.
*Pakistan*			
Alvarez E (2017)	Clinical Process Implementation	Treatment abandonment.	Over the course of the study, treatment abandonment rates decreased from 27% in 2001 to 7% in 2008. Rates of abandonment were highest in 2003 (pre-intervention) at 32%. These rates decreased after *medicina integral*, the interdisciplinary psychosocial team, began carrying out services for patients.
*Guatemala*			
Lima TM (2017)	Clinical Process Implementation	Adherence of women with inappropriate periodicity to colpocytological examination.	Adherence to colpocytological examination offered increased in the educational and behavioral intervention groups. Women in the behavioral group who received telephone reminders and scheduled appointments had greater levels of adherence (66.8%) than women in the educational group.
*Brazil*			
Mishra GS (2017)	Clinical Process Implementation	To create a triad chain of Creating Awareness, Early Detection and Rapid Diagnosis.	Over the course of the intervention, 3309 individuals with suspicious head and neck health findings were referred to tertiary care. Over half of those referred, 1890 (57.1%), were diagnosed with head and neck cancers. A majority of those referred 1712 (90.58%) began treatment. 343 defaulted on their treatment, which prompted health workers to visit them in their villages to restart treatment. Of those visited, 65 restarted treatment. 1434 (75.87%) completed the treatment process post-intervention.
*India*			
Riogi B (2017)	Clinical Process Implementation	Proportion of patients returning for follow-up at the breast clinic within 30 days.	There proportion of those who returned within a 30-day period in the navigated group was higher (57.9%) than in the non-navigated group (23.7%). The odds ratio [OR] was 4.43 [95% confidence interval, CI: 1.54–12.78]; p = 0.0026).
*Kenya*			
Vasconcelos CTM (2017)	Clinical Process Implementation	Rate of non-return to receive the pap test result after receiving any of the interventions.	A majority of women who received a pap test, 585 (75.5%), followed up to receive a result within 65 days. The group that received an educative session and test demonstration had the highest rates of return at 187/82.4%, while the behavioral group who received a recall ribbon had the lowest rates of return at 149/65.9%.
*Brazil*			
Chavarri Guerra Y (2018)	Clinical Process Implementation	Patients to obtain a referral to a cancer center and a specialist appointment within the first 3 months after enrollment in the program.	Nearly all patients (97%, 68) were navigated into a specialized cancer center for diagnosis or treatment. Of those referred, 91% of patients (95% CI 83%–96%) had a specialist appointment within the first 3 months after enrollment. Patient navigators conducted follow-up with each patient about 6 times during their time in the study.
*Mexico*			
Mireles-Aguilar T (2018)	Process Implementation	Broke down medical care barriers and reduced delays in accessing breast cancer care by assisting participants to schedule a medical consultation with a specialist.	A total of 446 medical consultations were scheduled, and 309 patients attended their appointments.
*Mexico*			
*Zi-Yi Y (2018)*	Clinical	Variances in diagnostic and treatment timeliness between navigated patients and patients diagnosed in the previous year.	Women who received patient navigation services received a timely mammography compared with patients in the prior year.
*Malaysia*			

## Discussion

As the need for patient navigation services for cancer care in LMICs becomes clearer, there is also a need to continually assess the effectiveness of these interventions and how they are implemented. This scoping review describes the published literature on cancer patient navigation interventions in LMICs. The research team identified fourteen studies that met our criteria for patient navigation interventions and outcomes in LMICs. Twelve of these articles reported on implementation and process outcomes, and nine reported on clinical outcomes. Although most of the articles reported on implementation outcomes, none of the articles reported the use of an implementation science framework in the design of their study. Patient navigation services were also offered among a diverse range of healthcare facilities from breast cancer care centers to hospitals to the workplace, but occurred mostly frequently at the tertiary care level. All 14 of these studies featured a first or senior author from an LMIC.

Patient navigation is thought to play an important role in reducing barriers to cancer care in high income countries (HICs) by increasing screening rates, access to early diagnosis and treatment adherence among other quality indicators [7–10, 12, 20]. Yet, few studies have focused on the role that patient navigation might play in cancer care in LMICs or on how these services can affect health outcomes in countries with fragmented or fragile health systems. In the 14 studies included in the review, the types of patient navigation services that were offered mirrored those provided in HICs, notably facilitating linkages to follow-up services [6, 31–35], providing counselling [6, 33, 34, 36–39], providing financial support [40], coordinating appointments [32, 33, 35, 39–43], and maintaining communication with patients and families [40, 41, 43].

All studies reported a positive impact of patient navigation interventions on the primary outcome of interest, but there was considerable variability among studies. Questions remain as to which types of interventions might effectively translate across resource settings and health systems contexts. Our review found that there are several gaps in the evidence on patient navigation for cancer care in LMICs that might affect the widespread applicability of findings. Despite the variety of services offered, only three articles explicitly called their intervention a “patient navigation” intervention. This has important implications for the way that patient navigation practices and policies might be framed in LMICs, and how strategies developed in HICs might be appropriately adapted to a given context. As the practice grows and researchers continue to explore the role of patient navigation in cancer care in LMICs, it may be valuable to develop a more contextualized definition of patient navigation to better capture and therefore address the needs of patients in different settings, as well as improve the provision of care. This new definition could provide a better framework for patient navigators in LMICs to ensure that patients receive seamless navigation from screening through survivorship.

The studies in this scoping review focused mainly on women’s cancers, predominantly breast and cervical cancer [6, 32, 34, 35, 37, 39–43], highlighting another gap in the evidence on patient navigation in cancer care. The over-representation of women’s cancers in this literature might be because breast and cervical cancer are among the most common cancer types in women in LMICs, and also because women often face additional social and/or cultural barriers [26, 44, 45] that can impact their opportunities to seek care for a new symptom, to be diagnosed at earlier stage of disease [46] and to complete their care pathway, from diagnosis through treatment and follow-up care.

The majority of articles included in this review focused on patient navigation services at the tertiary care level. In considering the challenges to accessing services such as lack of geographic access [5], sociocultural barriers and weak health systems [20, 26], limiting patient navigation services mostly at the tertiary level may contribute to a gap in cancer care, as most patients in LMICs are entering the health system at the primary care level. Navigation might play a more important role in improving cancer survival, if such strategies are employed at multiple points along the cancer care continuum. For example, in breast cancer, where early diagnosis and access to treatment improves an individual’s chance of survival [47], patient navigation may assist with raising community awareness, early diagnosis, access to treatment and facilitating patients completion of the care path.

While over half of the papers reported on all three types of outcomes (clinical, process and implementation), none of the outcomes demonstrated whether the patient navigation intervention increased the likelihood of survival or improved long-term health outcomes for the patient. For example, articles consistently reported on outcomes such as rates of follow up [6, 31, 33, 38–43], reach or uptake of the patient navigation intervention [6, 31, 38, 40–42], and treatment adherence and/or retention [37, 38, 40] and screening rates [38, 40, 41]. This has been a research challenge in HICs as well; data is limited regarding the impact of patient navigation interventions on cancer-specific survival [47, 48]. In the future, studies will need to consider the best ways to measure long-term clinical outcomes to provide new levels of validity to patient navigation interventions.

**Fig 2:**
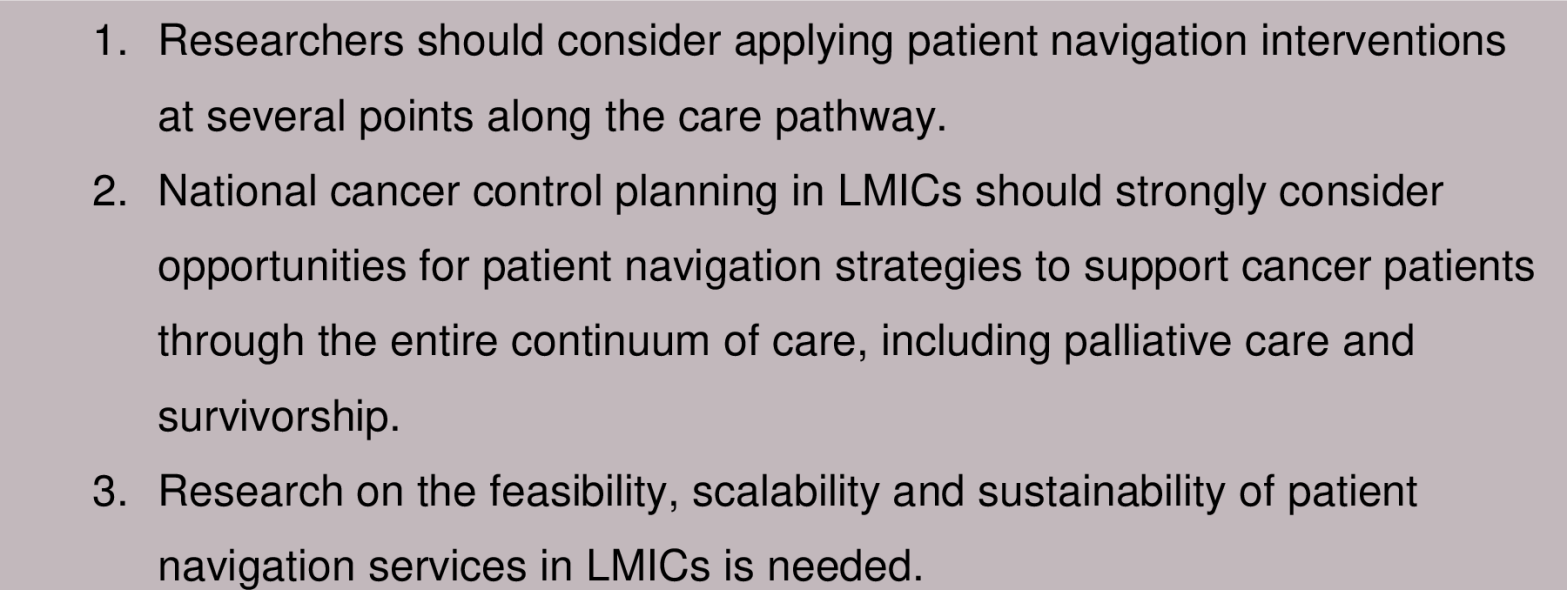
Recommendations for expanding patient navigation programs in LMICs.

This scoping review found that most studies discussed implementation research but did not explicitly mention this when describing their study design. The 14 studies we identified reported common implementation science outcomes, such as the uptake, acceptability, fidelity and feasibility of the interventions. This terminology suggests that researchers are conducting implementation science research but are not directly reporting this. Incorporating implementation science from the beginning may improve the measure of study outcomes and help elicit factors relevant to the scale- up of evidence-based interventions. Fig 2 describes suggested recommendations for health planners and researchers considering patient navigation programs in LMICs (Fig 2).

### Strengths

To our knowledge, this is the first scoping review of patient navigation services in cancer care in LMICs to be conducted.

#### Limitations

Although our search strategy was comprehensive, we may have failed to capture all studies that included patient navigation components in cancer care in LMICs. In particular, our search was restricted to publications in the English Language. As such, we likely omitted relevant publications from countries where the predominant language is a language other than English. Moreover, studies were only considered if they reported on outcomes directly related to patient navigation service provision. Lastly, to our knowledge, there is not an agreed upon definition of patient navigation in cancer care in LMICs.

## Conclusion

This scoping review provides a comprehensive overview of patient navigation interventions in cancer care in LMICs. The limited evidence from HICs suggests that providing patient navigation services in cancer care might lead to better patient outcomes [7–10, 13, 18]. Ensuring that patients receive the help they need to navigate the cancer care pathway is important to improving patient outcomes in LMICs, particularly in areas where access to healthcare is fragmented and health systems may be fragile and underfunded. The results from this scoping review suggest that providing patient navigation services in cancer care at multiple points along the cancer care pathway in LMICs can improve screening rates, post-operative complications and patient retention. Using implementation science frameworks [30, 49] from the outset of designing a patient navigation for cancer care intervention, may lead to better outcome measures. Developing a checklist to use when designing a patient navigation intervention for cancer care in LMICs would help to further the important role of patient navigation in cancer care in LMICs.

## Supporting information

S1 Table Systematic review search strategy. Search terms used to identify studies.
(DOCX)
